# BamBam: genome sequence analysis tools for biologists

**DOI:** 10.1186/1756-0500-7-829

**Published:** 2014-11-24

**Authors:** Justin T Page, Zachary S Liechty, Mark D Huynh, Joshua A Udall

**Affiliations:** Department of Biology, Brigham Young University, Provo, UT 84602 USA; Department of Plant and Wildlife Science, Brigham Young University, Provo, UT 84602 USA

**Keywords:** SAM, BAM, Haplotype, SNP, Indel, Consensus, Methylation, Expression

## Abstract

**Background:**

Massive computational power is needed to analyze the genomic data produced by next-generation sequencing, but extensive computational experience and specific knowledge of algorithms should not be necessary to run genomic analyses or interpret their results.

**Findings:**

We present BamBam, a package of tools for genome sequence analysis. BamBam contains tools that facilitate summarizing data from BAM alignment files and identifying features such as SNPs, indels, and haplotypes represented in those alignments.

**Conclusions:**

BamBam provides a powerful and convenient framework to analyze genome sequence data contained in BAM files.

**Electronic supplementary material:**

The online version of this article (doi:10.1186/1756-0500-7-829) contains supplementary material, which is available to authorized users.

## Findings

Massive amounts of data are involved in genome sequence research, requiring researchers to use supercomputing clusters and complex algorithms to analyze their sequence data. Genomic analyses frequently include next-generation sequencing to produce millions of short reads, followed by aligning of reads to a reference genome sequence with software like GSNAP and Bowtie 2 [1, 2]. These programs generate SAM files, the accepted standard for storing short read alignment data, which are subsequently compressed to BAM format via SAMtools [3]. The BAM files must then be analyzed and compared to produce meaningful results. Here we expand on the body of tools for analyzing and comparing BAM files.

We present BamBam, a package of bioinformatics tools to carry out a variety of genomic analyses on BAM files (Table 1). The included tools perform such tasks as counting the number of reads mapped to each gene in a genome (as for gene expression analyses), identifying SNPs (Single Nucleotide Polymorphisms) and CNVs (Copy Number Variants), and extracting consensus sequences. The purpose of BamBam is to provide a consistent framework to perform common tasks, without requiring extensive knowledge of computation or algorithms to select or interpret appropriate parameters.

**Table 1:** **The core independent tools of BamBam**

Section	Tool	Purpose
Single nucleotide Polymorphisms	InterSnp	Call SNPs between two or more samples
	Pebbles	Impute genotypes in output from InterSnp
	HapHunt	Phase haplotypes with K-means
Copy number Variants	GapFall	Identify deletions between two samples
	Elfen	Identify covered regions
	HMMph	Call copy number variants with HMM
Bisulfite-sequence Analysis	MetHead	Summarize base pair methylation in bisulfite-sequence data
GeneVisitor	Bam2Consensus	Generate consensus sequences from one or more samples
	Bam2Fastq	Extract mapped and unmapped reads from BAM files
	Counter	Summarize read coverage of sequences or regions
	SubBam	Extract subset of mapped reads
Allopolyploid analysis	PolyCat	Categorize reads by genome based on similarity to parents
Scripts	Various	Various

The BamBam package includes several independent programs, briefly described below. Brief tests were carried out to compare InterSnp, GapFall, and HapHunt with similar tools (Additional file 1). The latest version of PolyCat is also included [4]. The README in the download package provides example commands for various common analyses, including phylogeny inference, molecular evolution estimation, methylation analysis, and differential expression analysis. A usage guide (see Additional file 2) provides a more detailed walkthrough of some workflows.

### Single nucleotide polymorphisms

*InterSnp* calls SNPs between samples, represented by separate BAM files. InterSnp examines each position in the genome, assigning consensus alleles to each site for each sample. A SNP is called whenever two samples differ at the same position, producing a table with the genotypes of all samples at all polymorphic sites. The output is a table with the sequence name, position, and genotype for each sample at that site on each row, which can be readily processed by common command-line programs or scripts to calculate statistics or produce marker data for other programs.

*Pebbles* imputes genotypes using the K-nearest neighbor algorithm [5, 6]. For each unknown genotype, Pebbles finds the samples that are most similar at nearby loci. Then it assigns a genotype to the unknown locus based on the weighted contributions of those neighbors. Pebbles operates on InterSnp output—a table of genotypes—and produces a file of the same form.

**Figure 1:**
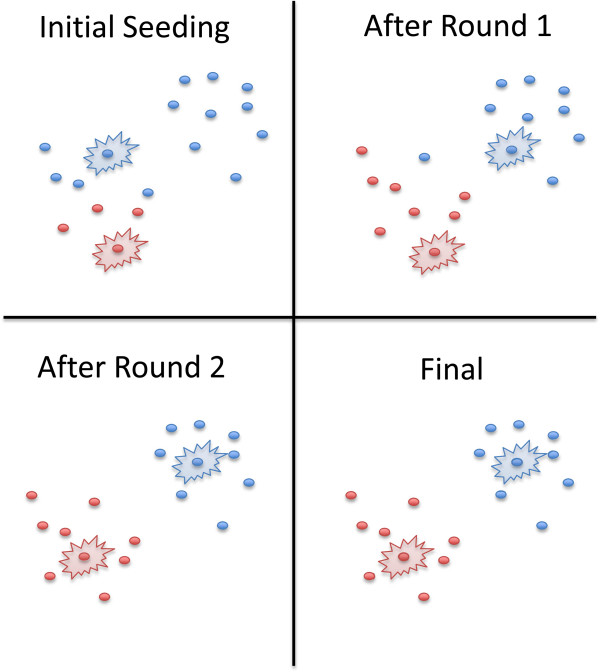
**K-means clustering algorithm.** An example 2-cluster run is shown, with the clusters distinguished by color and the current cluster seeds marked by a starburst. In the first round, each point is assigned to its closest seed, and a new seed is chosen for each cluster based on the average of all points in that cluster. As a result, the blue cluster seed moves to the right side. In the second round, both cluster seeds drift to their correct locations, resulting in a proper division. Note that, after two rounds, the clusters have reached a steady-state, and would not change further through an infinite number of iterations.

*HapHunt* uses K-means clustering to solve the haplotype-phasing problem, which consists of identifying all haplotypes in a sampled individual or population. Many programs have attempted to solve haplotype phasing and the closely related haplotype assembly problems using a variety of strategies, including Max-Cut, hidden Markov models, and dynamic programming [7–9]. The K-means clustering algorithm (Figure 1) is an unsupervised machine learning algorithm, and is mathematically equivalent to Principle Component Analysis [10, 11].

HapHunt first selects K reads distant from one another to serve as haplotype seeds. It assigns each other read to the haplotype with the closest consensus sequence. Then it recalculates the consensus sequences based on the reads in each haplotype and repeats the process of assigning each read to the haplotype with the closest consensus sequence. It repeats this process a given number of times, calculating a score at the end of each round based on the difference of the smallest interhaplotype distance and greatest intrahaplotype distance. This score favors clusterings in which haplotypes are individually compact and most distinct from one another. This score can optionally be scaled by the average size ratio for each pair of haplotypes, favoring clusterings that are more evenly divided. The consensus sequences of the final haplotypes are printed as an aligned FASTA file for each sequence in the original reference.

### Copy number variants

*Gapfall* identifies large deletions between samples based on read coverage. It searches the genome for extended regions that have high coverage in one sample but no coverage in the other. A large region with no coverage could indicate a physical deletion (for genomic samples) or a deactivated gene (for RNA-seq). These putative deletions are reported as an annotation file that can be visualized with a genome browser such as IGV [12].

*Eflen* identifies and extracts regions in a BAM file that are covered by at least a user-specified number of reads and outputs those regions as a GFF file. Provided with multiple BAMs, Eflen will identify regions that are covered in at least a user-specified fraction of those BAMs. This tool can be especially useful for analyzing GBS or RNA-seq data.

*HMMph* identifies CNVs between samples based on read coverage. BAM files must be provided for a control and for the sample of interest. The coverage ratio between those two BAM files is normalized by the total read coverage. Then the copy number of each locus in a sliding window is modeled based on a Poisson distribution in an untrained Hidden Markov Model [13, 14].

### Bisulfite-sequence analysis

**Figure 2:**
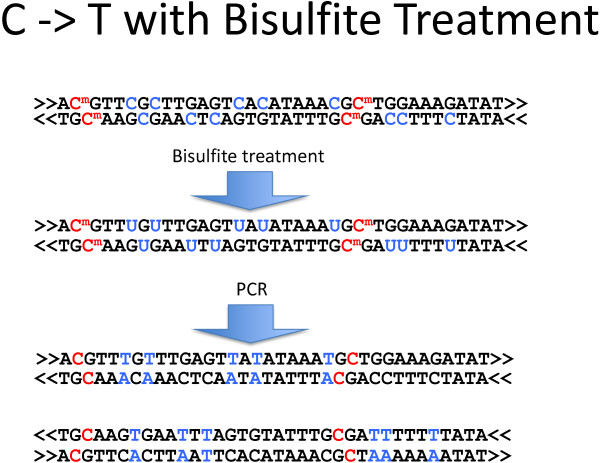
**Bisulfite treatment.** The effects of bisulfite treatment on DNA are shown. An “m” superscript indicates a methylated cytosine. The orientation of each strand is indicated by “<<” and “>>”. Bisulfite treatment converts unmethylated cytosines into uracils and, ultimately, thymines. After PCR, however, a given fragment may have C- > T conversions or G- > A conversions, depending on its orientation relative to its origin fragment.

Bisulfite treatment converts unmethylated cytosines to thymines. *MetHead* summarizes methylation at all cytosine positions in the genome, based on BAM files of mapped bisulfite-treated reads. It totals the number of mapped cytosines and thymines at each position (indicating methylated and unmethylated states, respectively), then performs a one-tailed binomial test for the methylation of that site.Different protocols are used for bisulfite treatment. If PCR is not performed after bisulfite treatment but before sequencing, then only 2 possibilities exist: conversions on the forward and reverse strand. But if PCR is performed, 4 possibilities exist (Figure 2). To properly count the number of cytosines and thymines in the 4-possibility protocol, the origin of the pre-PCR DNA fragment must be inferred. MetHead determines this—if necessary—by counting the number of C- > T conversions and G- > A conversions (indicative of a conversion on the reverse strand). It generates a BAM file with the orientation of each read matching its origin strand. That BAM can then be analyzed as if it were data produced by the 2-possibility protocol. Note that, in the produced BAM, the orientation of reads is not based on the direction in which the read was sequenced. Instead, the orientation of the read indicates the type of conversion caused by bisulfite treatment: C- > G or A- > T.

### GeneVisitor

It is often useful to be able to compute on specific genomic intervals, such as genes. GeneVisitor provides a quick and easy way to do this, using an annotation file (GFF or BED format) to call a function on each indicated region of the genome. This class can be used by C++ programmers to run custom functions. In addition, pre-built tools utilize GeneVisitor without the need for programming.

*Bam2Consensus* converts one or more BAM files into a series of FASTA-formatted consensus sequences. If desired, multiple sequences—essentially unphased haplotypes—can be produced per BAM file, facilitating analyses of heterozygosity, nucleotide diversity, and molecular evolution. Suppose you have several BAM files representing different accessions of a species, all mapped to a common genome reference sequence. With a single command, Bam2Consensus can produce an aligned FASTA file for each gene, each containing the consensus sequences for each accession.

*Bam2Fastq* extracts mapped or unmapped reads from a BAM file, or from select regions of the BAM file.

*Counter* summarizes the number of reads mapped to each annotated region in one or more BAM files. RPKM (Reads Per Kilobase per Million mapped reads) normalization can be applied if desired. The output of Counter is a table of features and read counts, ready to be imported into EdgeR for differential expression analysis [15].

*SubBam* extracts a subset of a BAM file. It can optionally modify the BAM file, changing the coordinates of mapped reads to match a new reference that is a subset of the original reference. Suppose you have WGS reads mapped to a reference sequence and are interested in several loci. SubBam can produce BAMs that only contain the loci of interest, with a coordinate system corresponding to the position in the locus, rather than in the genome as a whole.

### Allopolyploid analysis

**Figure 3:**
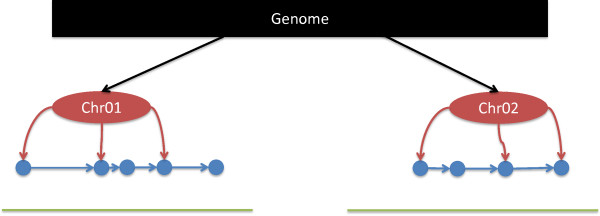
**Multi-level index.** The multi-level index provides random access to large numbers of individual base positions across a genome. Each sequence (green) is indexed by a linked-list (blue), and that index is indexed by a set of landmark nodes (red) to provide rapid access to any location.

The latest version of *PolyCat* is included in BamBam. PolyCat uses an index of known homoeo-SNPs (polymorphisms that distinguish the genomes of an allopolyploid) to identify the source genome for each read in a library, which cannot be distinguished through typical next-generation sequencing protocols [4].The MultiIndex class is used by PolyCat and MetHead, and can be used to make novel tools in C++. The MultiIndex is appropriate for random access to hundreds of millions of individual base positions in a genome sequence. It provides quick random access to base positions scattered across a genome sequence. Each sequence in the reference is indexed with a linked-list, with an index of landmark nodes spaced along the sequence at a resolution specified by the user (Figure 3).

### Scripts

In addition to the core tools mentioned above, BamBam includes many Perl scripts, many of which use BioPerl modules [16]. Script functions include calculation of nucleotide diversity (π) and molecular evolution rates (Ka and Ks), paralog identification, differential expression with EdgeR [15], summarization of results from MetHead, and summarization of genotype tables produced by InterSnp and Pebbles.

## Conclusions

The BamBam tools form a simple interface between the researching biologist and the wealth of data contained in next-generation sequence alignments. They provide a means to efficiently identify interesting genomic features and summarize data, facilitating many next-generation sequence analysis experiments.

BamBam is freely available under the MIT license at http://sourceforge.net/projects/bambam/. It depends on both SAMtools and BAMtools [3, 17].

## Availability and requirements

**Project Name:** BamBam

**Project Home Page:**http://sourceforge.net/projects/bambam/

**Operating System:** Unix

**Dependencies:** SamTools, BamTools, BioPerl

**Programming Language:** C++ and Perl

**License:** MIT

## Authors’ information

JP has a B.S. in Computer Science and is currently a graduate student in Biology, focusing on developing tools for polyploid genome analysis. ZL is an undergraduate student in the Udall lab. MH is a graduate student in the Udall lab. JU is an Associate professor at Brigham Young University and is the academic advisor of JP, ZL, and MH.

## Electronic supplementary material

Additional file 1:**Supplementary Material.** **Figure S1** Read alignment of cotton A-genome and d-genome reads to a common reference, rendered in IGV. Highlights indicate differences compared to the reference, so highlights in the upper sequence (A-genome) and the lack of those highlights in the lower sequence (D-genome) indicate SNPs between the two genomes. In this region, InterSnp identified 17 SNPs but SAMtools failed to identify any. **Figure S2** Haplotypes identified by SAMtools and HapHunt, compared to the known haplotype. **Figure S3** Phylogenetic tree. This neighbor-joining tree was built by neighbor based on SNPs identified by InterSnp. Then Geneious was used to render the actual tree. **Table S1** The number of deletions identified in each accession (row), along with the percentage of those deletions that were shared with other members of the same species and with the entire group of samples. (DOCX 207 KB)

Additional file 2:**BamBam User Guide**[15]**,**[18]**.**(DOCX 18 KB)
