# Supplementary material for: BamBam: genome sequence analysis tools for biologists
Source: BMC Res Notes. 2014 Nov 24;7:829. doi: 10.1186/1756-0500-7-829 (PMC4258253; doi:10.1186/1756-0500-7-829)
Supplement: Supplementary file 1 — Additional file 1:Supplementary Material. Figure S1 Read alignment of cotton A-genome and d-genome reads to a common reference, rendered in IGV. Highlights indicate differences compared to the reference, so highlights in the upper sequence (A-genome) and the lack of those highlights in the lower sequence (D-genome) indicate SNPs between the two genomes. In this region, InterSnp identified 17 SNPs but SAMtools failed to identify any. Figure S2 Haplotypes identified by SAMtools and HapHunt, compared to the known haplotype. Figure S3 Phylogenetic tree. This neighbor-joining tree was built by neighbor based on SNPs identified by InterSnp. Then Geneious was used to render the actual tree. Table S1 The number of deletions identified in each accession (row), along with the percentage of those deletions that were shared with other members of the same species and with the entire group of samples. (DOCX 207 KB) [file 13104_2014_3373_MOESM1_ESM.docx]

# Additional file 1 Supplementary Material

## InterSnp

We ran InterSnp and the SAMtools SNP caller on 2 BAM files, generated from the 2 genomes of an allotetraploid cotton species (SRA accession SRX204794) (Li, Handsaker, Wysoker, Fennell, & Ruan, 2009). In a 2000 bp region, InterSnp was able to identify 40 SNPs between the 2 individuals, which diverged ~10 mya and are expected to have several SNPs. In contrast, SAMtools identified only 5 SNPs, 1 of which was based on a single read from one of the individuals. Figure S1 shows a region of these alignments where InterSnp identified 17 SNPs and SAMtools found none. The specific SNPs identified obviously depend on the settings used with the two SNP callers. However, this example highlights a case where the default settings of SAMtools would not identify SNPs of interest to the user. Many users do not have sufficient knowledge of SAMtools to know which options to change.

While the SAMtools SNP caller is excellently suited for many analyses, it lacks the ability to compare individuals directly, i.e. it only detects differences relative to a single reference sequence. InterSnp’s ability to directly compare multiple individuals provides for massively parallel SNP detection and makes it ideal for many comparative genomic experiments.

## GapFall

The ChIP-seq analysis program MACS can perform a similar analysis to that of the BamBam program GapFall (Zhang et al., 2008). A test comparison was performed using sequence data from 3 accessions of 2 closely related cotton species: *Gossypium herbaceum* (A_1_) and *G. arboreum* (A_2_) (SRA project PRJNA202235). These 6 samples are closely related, so there should be few structural changes. MACS was run with default settings and a control sample from the more distant *G. raimondii*. GapFall was run with “-m3 –M 20” and the same *G. raimondii* control sample. The intersection of the different results was found with bedtools, and the fraction of gaps found in each test that was shared with the other members of that species (A_1_ or A_2_; Species) or with all accessions (A; Group) was calculated (Table S1). With one exception, GapFall found results that were more consistent than MACS across the same individuals. This is unsurprising, given that MACS is designed for ChIP-seq and looks for significant differences in coverage, whereas GapFall is looking for areas of zero or near-zero coverage, which MACS might simply ignore. GapFall, designed specifically to find long deletions, is more appropriate for that task. Laboratory-based confirmation of these deletions would provide a more robust comparison of these methods.

## HapHunt

We tested HapHunt by running it on the gene Gorai.001G0006400 from the *G. raimondii* reference sequence. We mapped reads from the allotetraploid (AD) *G. hirsutum cv. Maxxa* (SRR617482) to the diploid (D) *G. raimondii* reference. Thus, we expect the aligned reads to include representatives from both the A_T_- and D_T_-genomes of the tetraploid. Haplotypes were generated by SAMtools phase, HapHunt, and HapHunt with stacking (Li et al., 2009). Stacking means that several independent clusterings were performed, followed by a clustering of the results. We compared the putative haplotypes from each of these programs to reconstructed sequences for this gene, based on known homoeo-SNPs between the A_T_- and D_T_-genomes (Figure S2). An alignment of these 8 sequences shows that the putative haplotypes very closely matched the A_T_ and D_T_ sequences known and predicted by SAMtools. It’s noteworthy that the raw reads were only 100 bp long, but the gene was over 11,000 bp long, showing that HapHunt can accurately and consistently cluster haplotypes, without a need for reads that completely span the region. But in regions where low coverage and/or low SNP density separated adjacent SNPs, the phase of a single predicted sequence jumped from one haplotype to the other. HapHunt attempts to flag such cases with lowercase bases in the summary report, but it is an imperfect detector. As such, the phasing of a region may be incomplete around low SNP-density or low coverage. However, where there are sufficient SNPs to distinguish haplotypes and sufficient coverage to construct haplotypes across the region, HapHunt is capable of accurately phasing reads.

HapHunt demonstrates the algorithm for haplotype phasing by K-means clustering. It is based purely on sequence data contained in a BAM file, so no other information about the samples or their relationship is needed. While a higher degree of control over the details of the clustering algorithm is attainable through use of command-line options, HapHunt can be adequately run without tweaking and optimizing these parameters. Unlike other algorithms that assume diploid individuals and phase exactly 2 haplotypes, HapHunt can work on any number of haplotypes. Its performance on 2 haplotypes is similar to that of SAMtools phase, but HapHunt can also be applied to a polyploid or to a population that may have many more than 2 distinct haplotypes.

## References

Li, H., Handsaker, B., Wysoker, A., Fennell, T., & Ruan, J. (2009). The sequence alignment/map format and SAMtools. *…*.

Zhang, Y., Liu, T., Meyer, C. A., Eeckhoute, J., Johnson, D. S., Bernstein, B. E., et al. (2008). Model-based Analysis of ChIP-Seq (MACS), *9*(9), R137. doi:10.1186/gb-2008-9-9-r137

# Supplementary Figures

Figure S1 Read alignment of cotton A-genome and D-genome reads to a common reference, rendered in IGV. Highlights indicate differences compared to the reference, so highlights in the upper sequence (A-genome) and the lack of those highlights in the lower sequence (D-genome) indicate SNPs between the two genomes. In this region, InterSnp identified 17 SNPs but SAMtools failed to identify any.


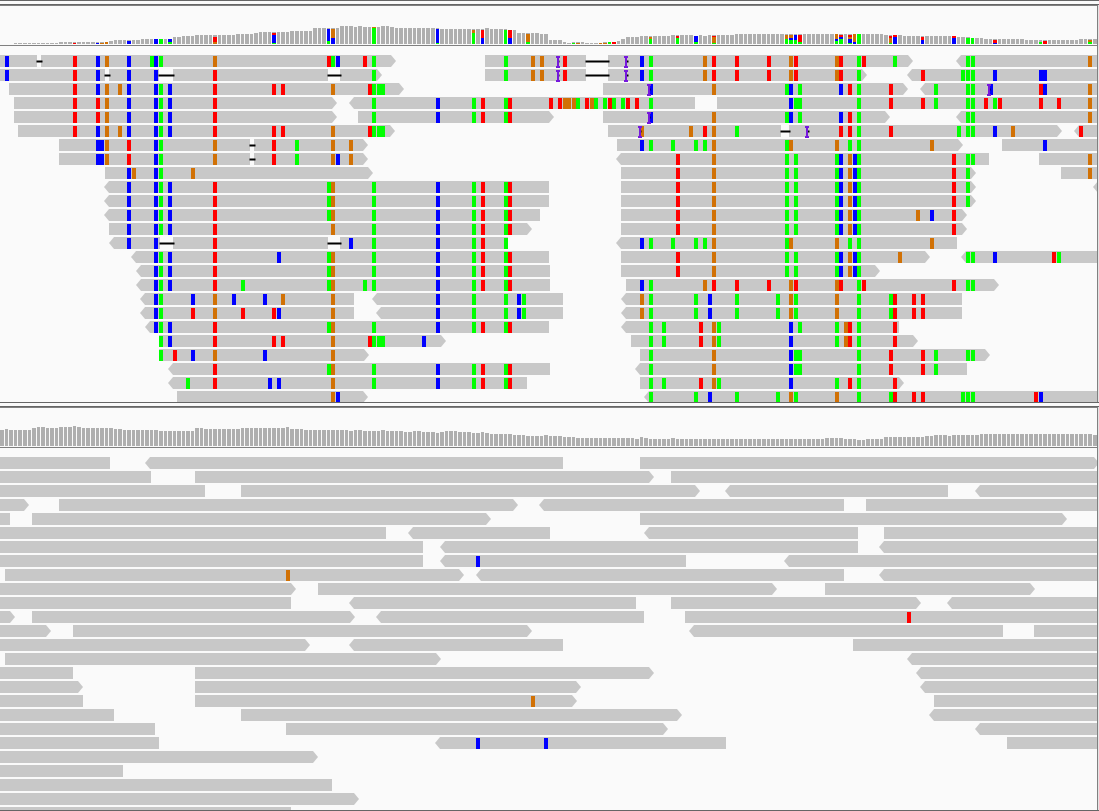


Figure S2 Haplotypes identified by SAMtools and HapHunt, compared to the known haplotype.

# Figure S3 Phylogenetic Tree. This neighbor-joining tree was built by neighbor based on SNPs identified by InterSnp. Then Geneious was used to render the actual tree.


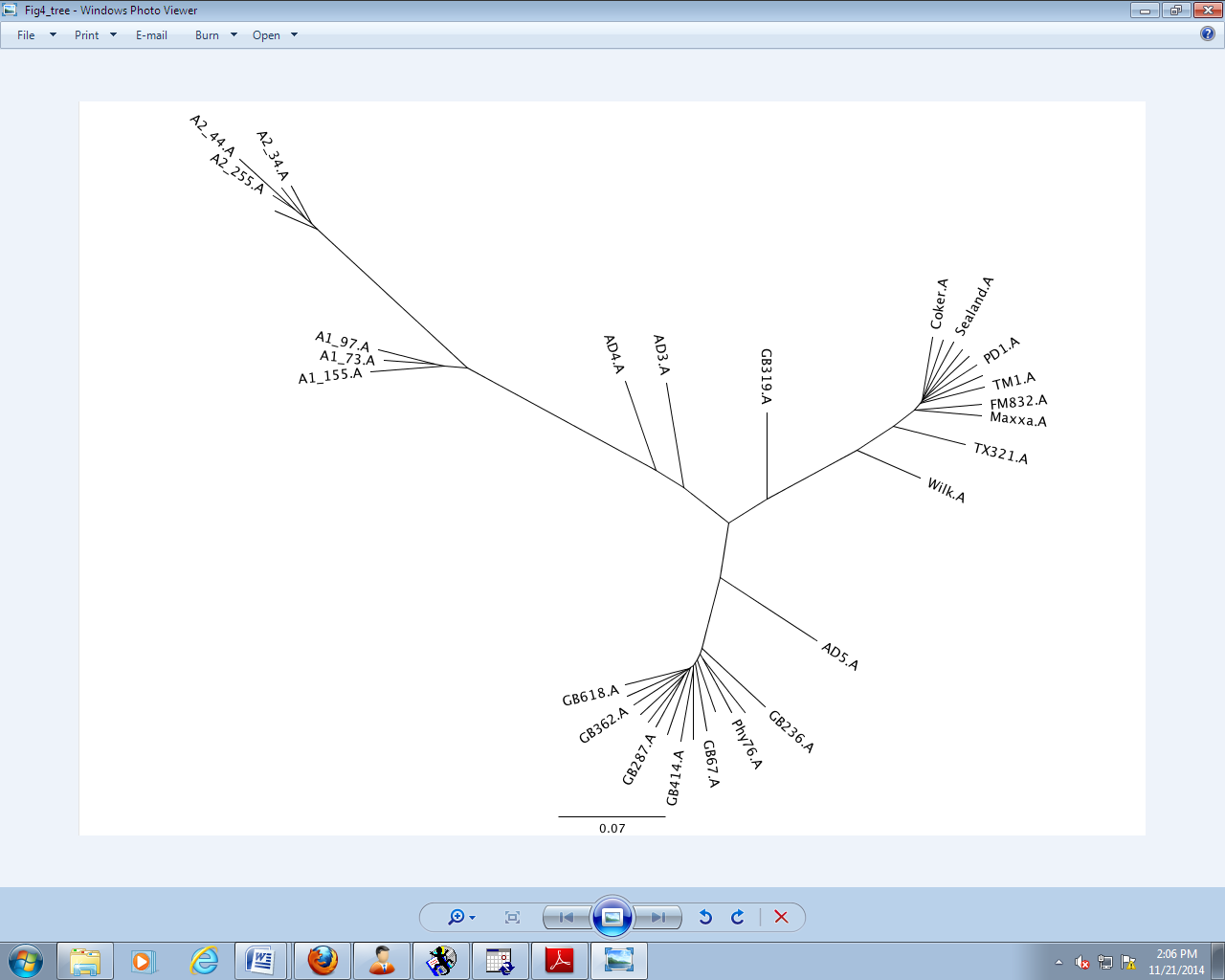


# Tables

Table S1 The number of deletions identified in each accession (row), along with the percentage of those deletions that were shared with other members of the same species and with the entire group of samples.

|  | **GapFall** | | | **MACS** | | |
| --- | --- | --- | --- | --- | --- | --- |
| **Accession** | **Total** | **Species** | **Group** | **Total** | **Species** | **Group** |
| A1-73 | 43,941 | 66.5% | 57.8% | 15,425 | 68.3% | 66.7% |
| A1-97 | 34,686 | 84.2% | 73.3% | 18,354 | 57.4% | 56.1% |
| A1-155 | 33,943 | 86.1% | 74.9% | 16,884 | 62.4% | 61.0% |
| A2-4 | 35,024 | 81.0% | 72.6% | 15,359 | 81.0% | 67.0% |
| A2-34 | 34,569 | 82.0% | 73.5% | 20,681 | 60.2% | 49.8% |
| A2-1011 | 33,588 | 84.4% | 75.7% | 21,247 | 58.5% | 48.4% |
