# Supplementary material for: BamBam: genome sequence analysis tools for biologists
Source: BMC Res Notes. 2014 Nov 24;7:829. doi: 10.1186/1756-0500-7-829 (PMC4258253; doi:10.1186/1756-0500-7-829)
Supplement: Supplementary file 2 — Additional file 2:BamBam User Guide[15],[18].(DOCX 18 KB) [file 13104_2014_3373_MOESM2_ESM.docx]

**Additional file 2: BamBam User Guide**

**File Formats**

The tools in BamBam operate primarily on BAM files, the standard format for sequence reads aligned to a reference genome. For a detailed explanation of this format, see <http://samtools.github.io/hts-specs/SAMv1.pdf> (or google “SAM format”). SAM and BAM files are generated by most read mapping software, and can be interconverted to each using samtools view. For example,

-bash-4.1$ samtools view -Sb mapped_reads.sam > mapped_reads.bam

BAM files must often be sorted and indexed. BamBam programs will specify if this is necessary for a given program. To sort,

-bash-4.1$ samtools sort mapped_reads.bam mapped_reads.sort

Once a BAM file has been sorted, it can be indexed as follows:

-bash-4.1$ samtools index mapped_reads.sort.bam

Indexing a BAM file produces a BAI file such as mapped_reads.sort.bam.bai, which must be kept with its corresponding BAM file (mapped_reads.sort.bam) if transferred to another location

Many BamBam tools also make use of annotation files in GFF and/or BED format. A GFF file has a line for each annotation, often genes or exons. Each line has 9 columns and looks like this:

Chr01 JGI gene 6158 6456 . - . Gorai.001G000100

A BED file is similar, but it has less columns.

Chr01 6158 6456 Gorai.001G000100

BamBam tools will sometimes use the 9^th^ column of a GFF, or the 4^th^ column of a BED, as the identifying name for that annotation in output reports.

**Installation**

To install BamBam, you must first install SAMtools (<http://www.htslib.org/>) and BAMtools (<https://github.com/pezmaster31/bamtools>). Then download the latest BamBam tarball from (<https://sourceforge.net/projects/bambam/>). Then do the following (change file name “1.2” to reflect the most recent version):

-bash-4.1$ tar xzf bambam-1.2.tgz

-bash-4.1$ cd bambam

-bash-4.1$ make all

All BamBam executables should now be located in bambam/bin. For instructions and usage information for each program, simply run it without providing any arguments. This will also work for the perl scripts in bambam/scripts.

**Calling SNPs and Building Phylogenies**

InterSnp calls SNPs between individuals (NOT relative to a reference sequence). You give InterSnp 2 or more BAM files, which must be sorted and indexed. The command looks likes this, using the -m option to require 10 reads to call an allele:

-bash-4.1$ interSnp -m 10 A.bam B.bam C.bam > AvsBvsC.snp

It will generate output that looks like this:

#Chr Pos A.bam B.bam C.bam

Chr01 2633 C A A

Chr01 2639 C A A

Chr01 2640 A G A

Chr01 2647 A G G

Chr01 2678 G N C

This is called a SNP file, and it has one output line per polymorphic site. An “N” means that there was not sufficient coverage to call a genotype for that individual at that site. Several tools and scripts in BamBam will make use of these SNP files.

InterSnp is able to process hundreds of samples in a single run (for example, in genotyping-by-sequencing). In these cases, you will often have a lot of Ns in your SNP file. You can attempt to fill in some of these missing genotypes by imputation, using the program Pebbles. With pebbles, use -m to indicate the minimum number of individuals that must have a given allele and -k to indicate the number of neighbors used to impute a missing genotype:

-bash-4.1$ pebbles -m 5 –k 5 AvsBvsC.snp > AvsBvsC.imputed.snp

The output will have the same format as the SNP file. A simple way to build a phylogeny from these data is to generate a distance matrix as follows (this uses a perl script from bambam/scripts):

-bash-4.1$ snp2dist.pl AvsBvsC.imputed.snp > AvsBvsC.dist

This distance matrix is the input to the PHYLIP program “neighbor”, and looks like this:

3

A.bam 0 0.388279558488389 0.398409570350586

B.bam 0.388279558488389 0 0.0334506301646224

C.bam 0.398409570350586 0.0334506301646224 0

If you have PHYLIP installed, you can then build a neighbor-joining tree using the following:

-bash-4.1$ cp AvsBvsC.dist infile

-bash-4.1$ neighbor

Now the file “outtree” contains a tree in newick format. You can open such a tree in any tree-viewing software, such as Geneious (Figure S3)[18].

**Calling Differential Gene Expression**

If your mapped reads are RNA-seq, then you can use Counter to generate a table of gene expression values. You can provide an annotation file (see “File Formats”) with the -g option, as follows:

-bash-4.1$ counter -g gene.gff A.bam B.bam C.bam > counts.txt

This will generate a table of gene expression values, like this:

gene A.bam B.bam C.bam

Gorai.001G000200 0 0 0

Gorai.001G000400 143 84 54

Gorai.001G000500 350 257 229

Gorai.001G000800 185 175 173

Gorai.001G000900 552 480 396

Gorai.001G001000 187 144 103

Gorai.001G001100 680 603 596

Gorai.001G001200 708 564 542

Gorai.001G001300 1112 885 738

If you want to normalize according to RPKM, just add “-n 1” to your counter command. The output file can be imported into the statistical sofwareR and analyzed for differential gene expression using a package like EdgeR [15].

**Analyzing Methylation**

MetHead can summarize methylation across the genome. First, you’ll need to prepare an index of cytosines with fasta2cyt.pl (in bambam/scripts):

-bash-4.1$ fasta2cyt.pl cotton.fasta > cotton.cyt

Then provide MetHead with both the reference sequence and the cytosine index, using the appropriate options.

-bash-4.1$ metHead -r cotton.fasta -c cotton.cyt A.bam > A.meth

The output is a tab-delimited file like the following:

Chr01 3996 CHH+ 3 2 0.917593

Chr01 4012 CHG- 5 2 0.722001

Chr01 4015 CHH+ 5 2 0.722001

Chr01 4018 CHH+ 5 2 0.722001

Chr01 4023 CHH- 6 2 0.608310

Chr01 4033 CHH+ 3 2 0.917593

Chr01 4037 CHH+ 3 2 0.917593

Each line corresponds to a single cytosine in the genome. The first two columns are the reference sequence and position. The third is the context, with a + or – to indicate the strand. Column 4 is the number of mapped cytosines (i.e., methylated votes) and column 5 is the number of mapped thymines (i.e., unmethylated votes). Column 6 is the p-value result of a binomial test for methylation. Thus, a p-value < 0.05 indicates that a cytosine is methylated.
